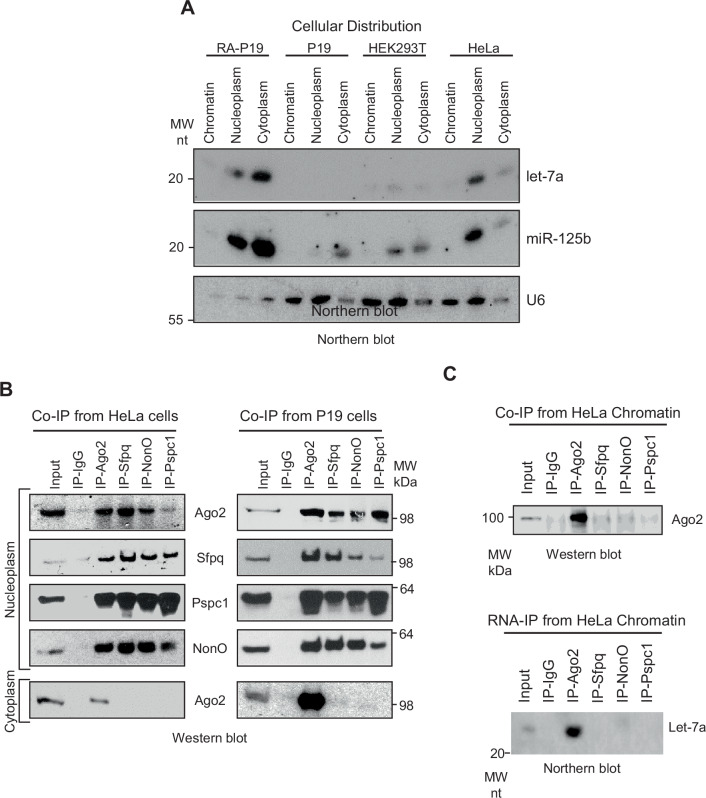# Addendum: Post-transcriptional gene silencing mediated by microRNAs is controlled by nucleoplasmic Sfpq

**DOI:** 10.1038/s41467-026-69354-8

**Published:** 2026-04-17

**Authors:** Silvia Bottini, Nedra Hamouda-Tekaya, Raphael Mategot, Laure-Emmanuelle Zaragosi, Stephane Audebert, Sabrina Pisano, Valerie Grandjean, Claire Mauduit, Mohamed Benahmed, Pascal Barbry, Emanuela Repetto, Michele Trabucchi

**Affiliations:** 1 INSERM U1065, C3M, Team Control of Gene Expression (10), 151 route de St-Antoine-de-Ginestière, B.P. 2 3194, Nice, 06204 France; 2 Université Côted’Azur, INSERM, C3M, 151 route de St-Antoine-de-Ginestière, B.P. 2 3194, Nice, 06204 France; 3 Université Côte d’Azur, CNRS, IPMC, Valbonne, France; 4 CRCM, Marseille Protéomique, Institut Paoli-Calmettes, Aix Marseille University, INSERM, CNRS, 27 bd Leï Roure, BP 30059, Marseille, 13273 France; 5 Université Côte d’Azur, CNRS, INSERM, IRCAN, Faculty of Medicine, 28 Av. Valombrose, Nice, 06107 France; 6 Université Lyon 1, UFR Médecine Lyon Sud, Lyon, F-69921 France; 7 Hospices Civils de Lyon, Hopital Lyon Sud, Laboratoire d’Anatomie et de Cytologie Pathologiques, Pierre-Bénite, F-69495 France; 8 Centre Hospitalier Universitaire de Nice, Département de Recherche Clinique et d’Innovation, Nice, F-06001 France

Addendum to: *Nature Communications* 10.1038/s41467-017-01126-x, published online 30 October 2017

Following the identification of an error in the original article, the authors have corrected Figure 5d and Supplementary Fig. 4c in this Addendum and amended the ‘Cell Fractionation’ subsection of the Method section together with its references. In the original publication, Gagnon *et al*. (ref ^67^ in the original article) was cited as the protocol used for all cell fractionation experiments. However, in Figure 5d Core *et al*. (ref ^68^ in the updated article) was used, whereas in Supplementary Fig. 4c Minajigi *et al*. (ref ^69^ in the updated article) was used for the chromatin solubilization step, both without citation.

The original Cell Fractionation section of the Methods read as:

“Cellular fractionation of cytoplasm, nucleoplasm, and chromatin was performed as previously described (ref ^67^), with a modification for the chromatin solubilization according to a previous protocol. Briefly, genomic DNA pellet was resuspended for treatment with Turbo DNase I in the DNase I digestion buffer (50 mM Tris pH 7.5, 0.5% Nonidet-P 40, 0.1% sodium lauroyl sarcosine, 1× Complete protease inhibitors) at 37 °C for 45 min with intermittent vortexing. The genomic DNA was further solubilized by adding 1% SDS, 0.3 M lithium chloride, 25 mM EDTA, and 25 mM EGTA and incubated at 37 °C for 15 min.”

The authors have repeated the experiments in question using the Gagnon *et al*. (ref ^67^) protocol for Figure 5D, and the Gagnon *et al*. (ref ^67^) protocol with Minajigi *et al*. (ref ^69^) for the chromatin solubilization step for Supplementary Fig. 4c. Specifically, the chromatin pellet was resuspended in 50 mM Tris-HCl (pH 7.5), 0.5% NP-40, and 0.1% sarkosyl, then sonicated, treated with DNase I, and supplemented with LiCl (final 300 mM) and 1% sarkosyl. Protease inhibitor cocktail (Roche) and RNaseOut (for RNA analyses) were included in all buffers.

The Methods section has been updated as:

“Cellular fractionation of cytoplasm, nucleoplasm, and chromatin was performed as previously described (ref ^67^), with a modification for the chromatin solubilization according to a previous protocol. Briefly, genomic DNA pellet was resuspended for treatment with Turbo DNase I in the DNase I digestion buffer (50 mM Tris pH 7.5, 0.5% Nonidet-P 40, 0.1% sodium lauroyl sarcosine, 1× Complete protease inhibitors) at 37 °C for 45 min with intermittent vortexing. The genomic DNA was further solubilized by adding 1% SDS, 0.3 M lithium chloride, 25 mM EDTA, and 25 mM EGTA and incubated at 37 °C for 15 min.

For IP experiments the salinity of 300 μg of protein lysate from cytoplasmic, nucleoplasmic, or genomic DNA solutions was adjusted to 150 mM of NaCl.

Cellular fractionation for Figure 5D was performed according to the protocol of Core *et al*. (ref ^68^). For Supplementary Fig. 4c, fractionation followed the method of Gagnon *et al*. (ref ^67^), with the chromatin solubilization step adapted from Minajigi *et al*. (ref ^69^). Specifically, the chromatin pellet was resuspended in 50 mM Tris-HCl (pH 7.5), 0.5% NP-40, and 0.1% sarkosyl, then sonicated, treated with DNase I, and supplemented with LiCl (final 300 mM) and 1% sarkosyl. Protease inhibitor cocktail (Roche) and RNaseOut (for RNA analyses) were included in all buffers.”

The source data, available online in https://figshare.com/s/b5c94f43d01972ff23f0, was provided to the Journal and independently assessed by an external expert. The versions of Figure 5 d and Supplementary Fig. 4c repeated using Gagnon *et al*. (ref ^67^) and Minajigi *et al*. (ref ^69^) are shown below. The updated *Methods* section that cites Core *et al*. (ref ^68^), Gagnon *et al*. (ref ^67^), and the chromatin solubilization step from Minajigi *et al*. (Ref. ^69^), has been updated in both the HTML and pdf version of the article along with the *References* section. This change does not affect the results, interpretation, and conclusions of the paper. All authors agree to the publication of this Addendum except for Dr Matégot. The authors would like to acknowledge the contribution of Dr. Roberta Veglia Tranchese in conducting the experiments for this Addendum.

Incorrect figure 5
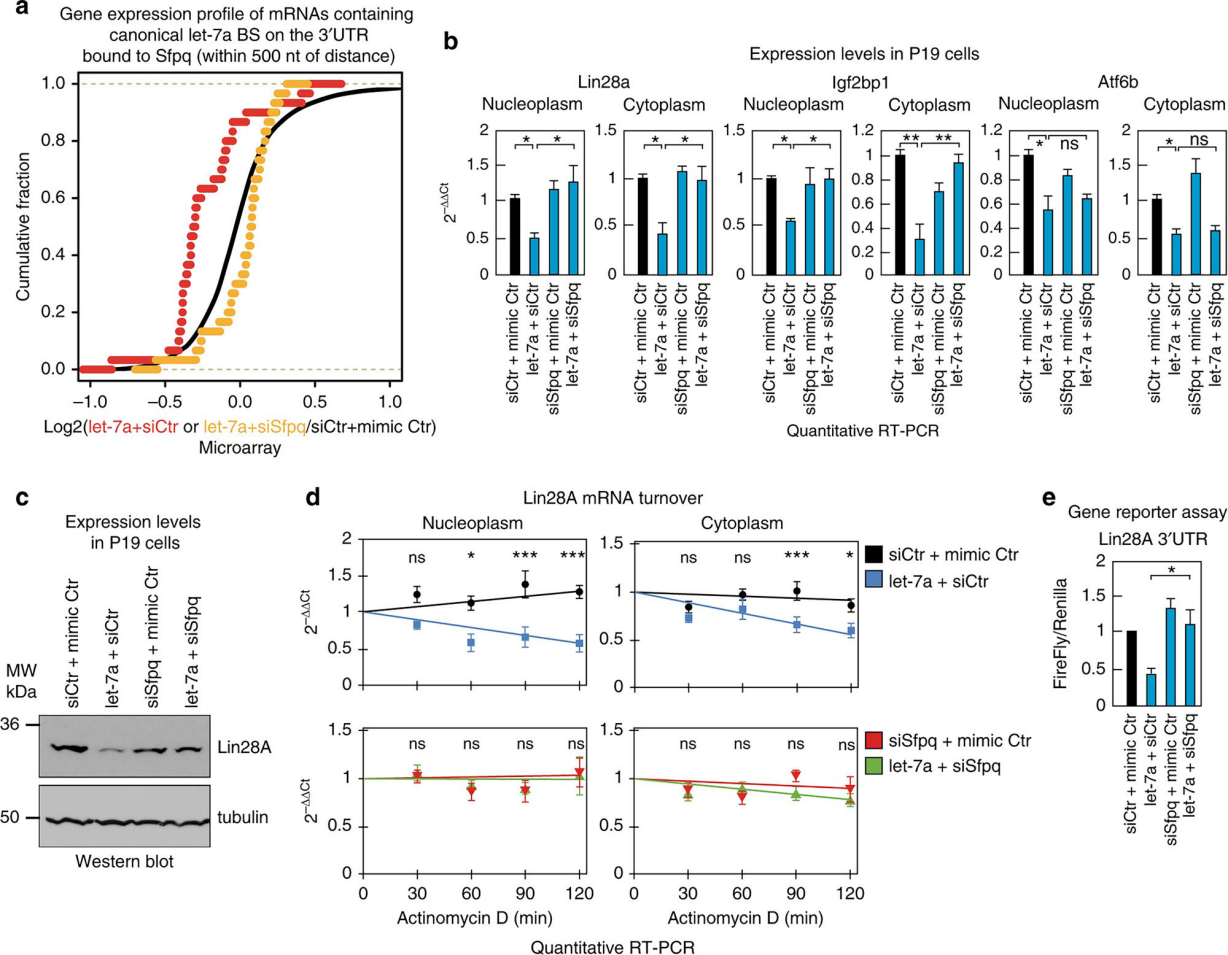


Corrected figure 5
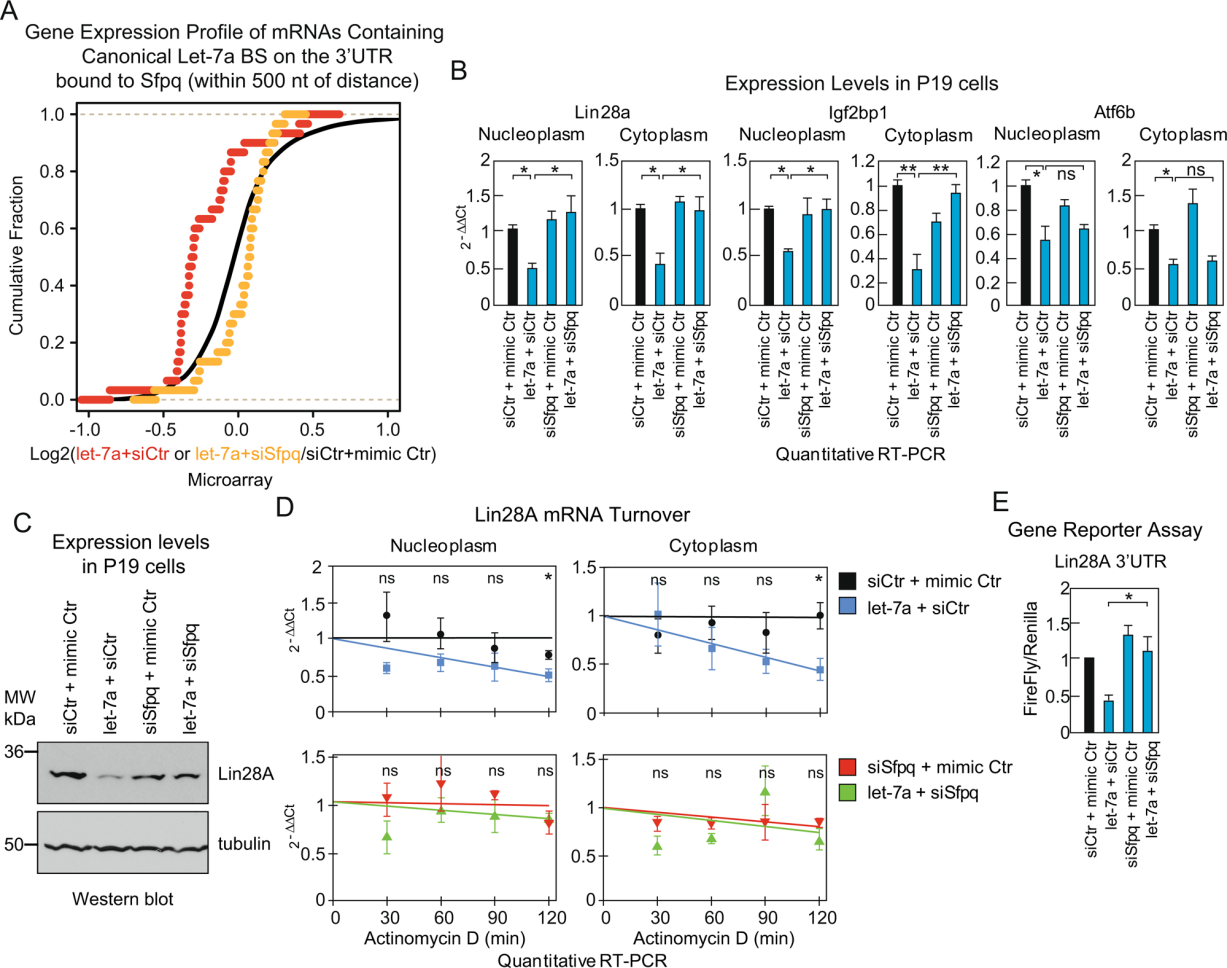


Incorrect supplementary figure 4
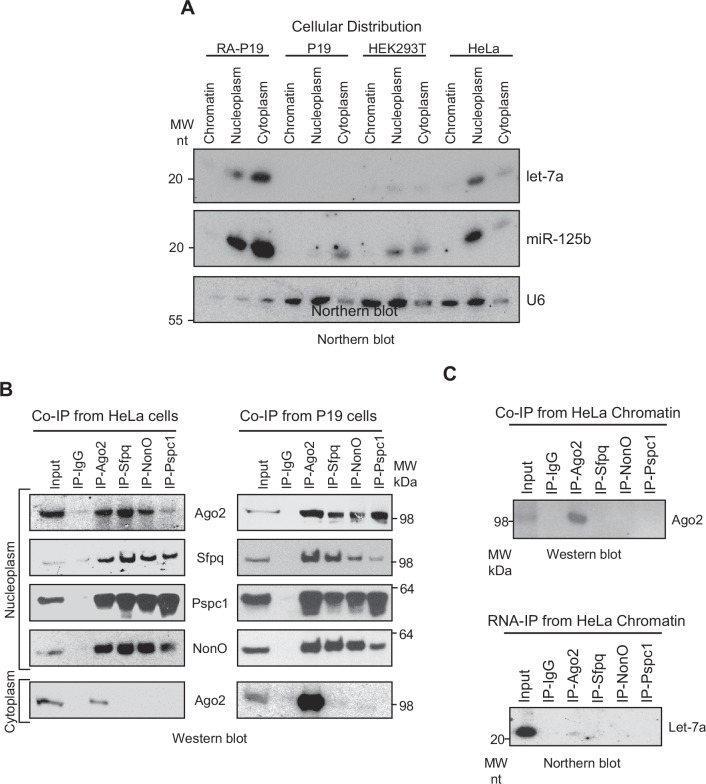


Corrected supplementary figure 4