# Supplementary material for: Post-transcriptional gene silencing mediated by microRNAs is controlled by nucleoplasmic Sfpq
Source: Nat Commun. 2017 Oct 30;8:1189. doi: 10.1038/s41467-017-01126-x (PMC5662751; doi:10.1038/s41467-017-01126-x)
Supplement: Supplementary file 1 — Supplementary Information [file 41467_2017_1126_MOESM1_ESM.pdf]

## Supplementary Information

### Supplementary Table 1

**Oligonucleotides used for quantitative RT-PCR (RT-qPCR), cloning, EMSA, and Northern blot analyses.**

| | Forward primer | Reverse primer |
|---|---|---|
| let-7a (qPCR) | 5'-TGAGGTAGTAGGTTGTATAGTT-3' | |
| miR-24 (qPCR) | 5'-TGGCTCAGTTCAGCAGGAACAG -3' | |
| miR-125b (qPCR) | 5'-TCCCTGAGACCCUAACTTGTGA -3' | |
| universal primer (qPCR) | | Universal primer from N-code kit (Life-Technology) |
| Mouse U2 snRNA(qPCR) | 5'-GAAGTAGGAGTTGGAATAGGA -3' | 5'-ACCGTTCCTGGAGGTACTG -3' |
| Human/Mouse U6 snRNA (qPCR) | 5'- CGCTTCGGCAGCACATATAC -3' | 5'- AAATATGGAACGCTTCACGA -3' |
| Human U2 snRNA(qPCR) | 5'- GAGCAGGGAGATGGAATAGGA -3' | 5'-ACCGTTCCTGGAGGTACTG -3' |
| Human Phc3 - miR-302b binding site (qPCR) | 5'- TGTTGTCCCCACTCAAAACA-3' | 5'-TGTGATAGCAAACAAGCCATTT-3' |
| mouse Phc3 - miR-302b binding site (qPCR) | 5'- CCTCCAGCAATAATGCTTCC -3' | 5'- CACGATGGGCTGGTTTTAGT -3' |
| Human Phlpp2 - miR-302b binding site (qPCR) | 5'- TGAGAGCACTTTTCATTGACTG -3' | 5'- GATTGGCTCGAGTGCAGATT -3' |
| mouse Phlpp2 - miR-302b binding site (qPCR) | 5'- CAGAACCAATCAAGCAGGTG -3' | 5'- TCCGAGTGCAGATTCAAAAA -3' |
| Human Crtc2 - miR-302b binding site (qPCR) | 5'- GGAGGGCCTAAAGCACTTGT -3' | 5'- TCCAGACAGACGGTTCAAAG -3' |
| mouse Crtc2 - miR-302b binding site (qPCR) | 5'- GGAGGGCCTAAAAGCACTTG -3' | 5'- AGGCTCTGACCTCCAGACAG -3' |
| mouse Hic2 (qPCR) | 5'- GCAAGGCCTGTAAAGACTGG -3' | 5'- GGAAGACCTTGGTCCCTTTC -3' |
| mouse Mier2 (qPCR) | 5'- TCCATGCCCAGTACCACATA -3' | 5'- CTCCACCTCCCAAGTGCTTA -3' |
| mouse Lin28a – let-7a binding site (qPCR) | 5'- TGAGTATGGGCCTCAGTGG -3' | 5'- ACACACCACCCAATGTGTTC-3' |
| mouse Igf2bp1 – let-7a binding site (qPCR) | 5'- GGATGAACTACCTCAGTCCTC -3' | 5'- AGGCATGAGTCCCTGCCCTC -3' |
| mouse Dusp1 – let-7a binding site (qPCR) | 5'- AAGAACCAAATACCTCAATT -3' | 5'- GGGTCTATTTACAAGATAGT -3' |
| mouse Atf6b – let-7a binding site (qPCR) | 5'- CCCGAGGGTACCTCGTGAGG -3' | 5'- GATAAAGGAGAGAATGAAGA -3' |

| | | |
|---|---|---|
| human Lin28a – let-7a binding site (qPCR) | 5’- GCACAGCCTATTGAACTACCTC -3’ | 5’- AGAAAAGCCAGCTCTTATTGG -3’ |
| mouse beta-2 microglobulin (qPCR) | 5’- CTGCTACGTAACACAGTTCCACCC-3’ | 5’-CATGATGCTTGATCACATG-3’ |
| mouse actin b (qPCR) | 5’- CGGTTCCGATGCCCTGAGGCTCTT -3’ | 5’- CGTCACACTTCATGATGGAATTGA -3’ |
| GFP (qPCR) | 5’- AGGTGAAGTTCGAGGGCGAC -3’ | 5’- TTGTACTCCAGTTGTGCCC -3’ |
| firefly (qPCR) | 5’- GGAACAACTTTACCGACCGC -3’ | 5’- TTCCATCTTCCAGGGATACG -3’ |
| Mouse Lin28 mRNA (RT-qPCR and NRO) | 5’- GTTCGGCTTCCTGTCTATGA -3’ | 5’- GTTGTAGCACCTGTCTCCTT -3’ |
| Sfpq binding sites Motif#1 and #2 n1 (EMSA) | 5'- CUGUACUGUA -3' | |
| Sfpq binding sites Motif#1 n2 (EMSA) | 5'- CUGACUGA -3' | |
| Sfpq binding sites Motif#2 n1 (EMSA) | 5'- UUGUAUUGUA -3' | |
| Sfpq binding sites Motif#2 n2 (EMSA) | 5'- AUGUAAUGUA -3' | |
| Sfpq binding sites Motif#2 n3 (EMSA) | 5'- GUGUAGUGUA -3' | |
| Sfpq negative binding site RNA oligo (EMSA) | 5’- CCCCGCCCCG -3’ | |
| Human Sfpq- RRM1 and 2 domains (cloning) | 5’- GGAGAGAAAACTTACACAC -3’ | 5’- CTAATGATGATGATGATGATG-3’ |
| let-7a (Northern blot) | | 5’-AACTATACAACCTACTACCTCA -3’ |
| miR-23b (Northern blot) | | 5’- GGTAATCCCTGGCAATGTGAT-3’ |

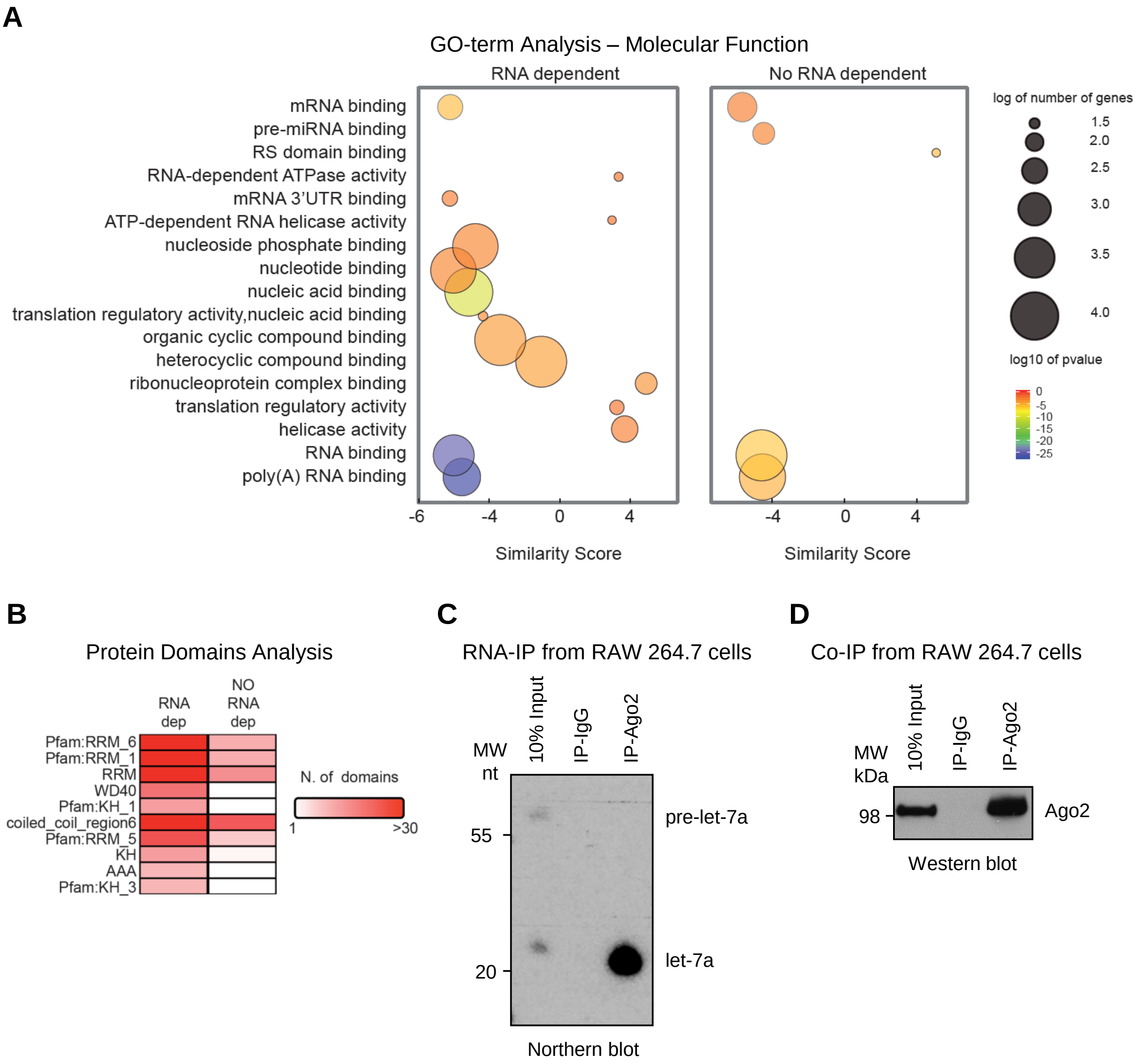
A
GO-term Analysis – Molecular Function
RNA dependent
No RNA dependent
mRNA binding
pre-miRNA binding
RS domain binding
RNA-dependent ATPase activity
mRNA 3'UTR binding
ATP-dependent RNA helicase activity
nucleoside phosphate binding
nucleotide binding
nucleic acid binding
translation regulatory activity,nucleic acid binding
organic cyclic compound binding
heterocyclic compound binding
ribonucleoprotein complex binding
translation regulatory activity
helicase activity
RNA binding
poly(A) RNA binding
-6
-4
0
4
Similarity Score
log of number of genes
1.5
2.0
2.5
3.0
3.5
4.0
log10 of pvalue
0
-5
-10
-15
-20
-25
B
Protein Domains Analysis
RNA dep
NO RNA dep
Pfam:RRM_6
Pfam:RRM_1
RRM
WD40
Pfam:KH_1
coiled_coil_region6
Pfam:RRM_5
KH
AAA
Pfam:KH_3
N. of domains
1
>30
C
RNA-IP from RAW 264.7 cells
MW nt
10% Input
IP-IgG
IP-Ago2
55
20
pre-let-7a
let-7a
Northern blot
D
Co-IP from RAW 264.7 cells
MW kDa
10% Input
IP-IgG
IP-Ago2
98
Ago2
Western blot

**Supplementary Figure 1. Proteomic analysis to identify RNA-dependent proteins interacting to Ago2.** (A) GO Term analysis of RNA–dependent and –independent interactors of Ago2 found by MS analysis. Scores among the enriched GO TERM were calculated by REVIGO [1]. Color code and different radius of circle meaning are indicated. (B) Protein domain enrichment on RNA–dependent and –independent interactors of Ago2 found by MS analysis. Color code is indicated. (C) RNA-IP of let-7a with Ago2. RAW 264.7 cell extracts were immunoprecipitated with anti-Ago2 antibody, RNA was purified, and analyzed by Northern blotting. (D) Immunoprecipitation of endogenous Ago2 in RAW 264.7 cells and immunoblotted with anti-Ago2 antibody.

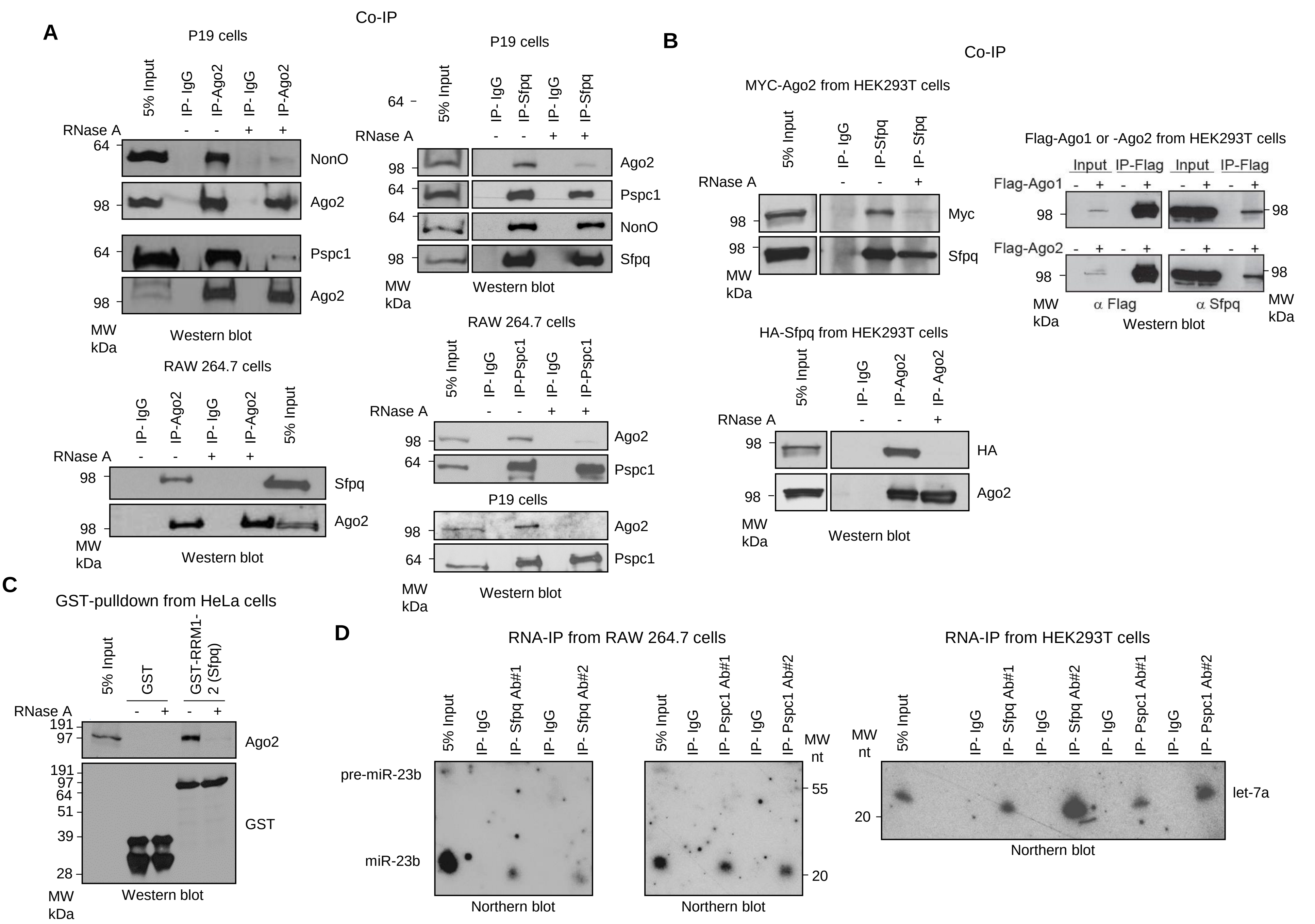
A
Co-IP
P19 cells
5% Input
IP- IgG
IP-Ago2
RNase A
NonO
Ago2
Pspc1
MW kDa
Western blot
RAW 264.7 cells
Sfpq
IP-Sfpq
NonO
IP-Pspc1
P19 cells
B
MYC-Ago2 from HEK293T cells
IP- Sfpq
Myc
HA-Sfpq from HEK293T cells
HA
Flag-Ago1 or -Ago2 from HEK293T cells
Input
IP-Flag
Flag-Ago1
Flag-Ago2
α Flag
α Sfpq
C
GST-pulldown from HeLa cells
GST
GST-RRM1-2 (Sfpq)
D
RNA-IP from RAW 264.7 cells
IP- Sfpq Ab#1
IP- Sfpq Ab#2
pre-miR-23b
miR-23b
IP- Pspc1 Ab#1
IP- Pspc1 Ab#2
MW nt
Northern blot
RNA-IP from HEK293T cells
let-7a

**Supplementary Figure 2. Sfpq, Pspc1, and NonO interact with the miRISC.** (A) Co-IP of the indicated endogenous proteins in the indicated cell lines. (B) Co-IP of the indicated proteins from HEK293T cells. Cells were transfected with the indicated tagged-proteins. (C) GST-pulldown experiment from 500 μg proteins from HeLa cell extract. Pulldown was performed with either the two RRM domains from human Sfpq fused with GST or GST alone as control. (D) RNA-IP of the indicated miRNAs with the endogenous Sfpq and Pspc1 from the indicated cell lines. Cell extracts were immunoprecipitated with two different antibodies and RNA was purified and analyzed by Northern blotting. When indicated, cell lysates were incubated at room temperature with RNase A (10 mg ml^-1) for 30min.

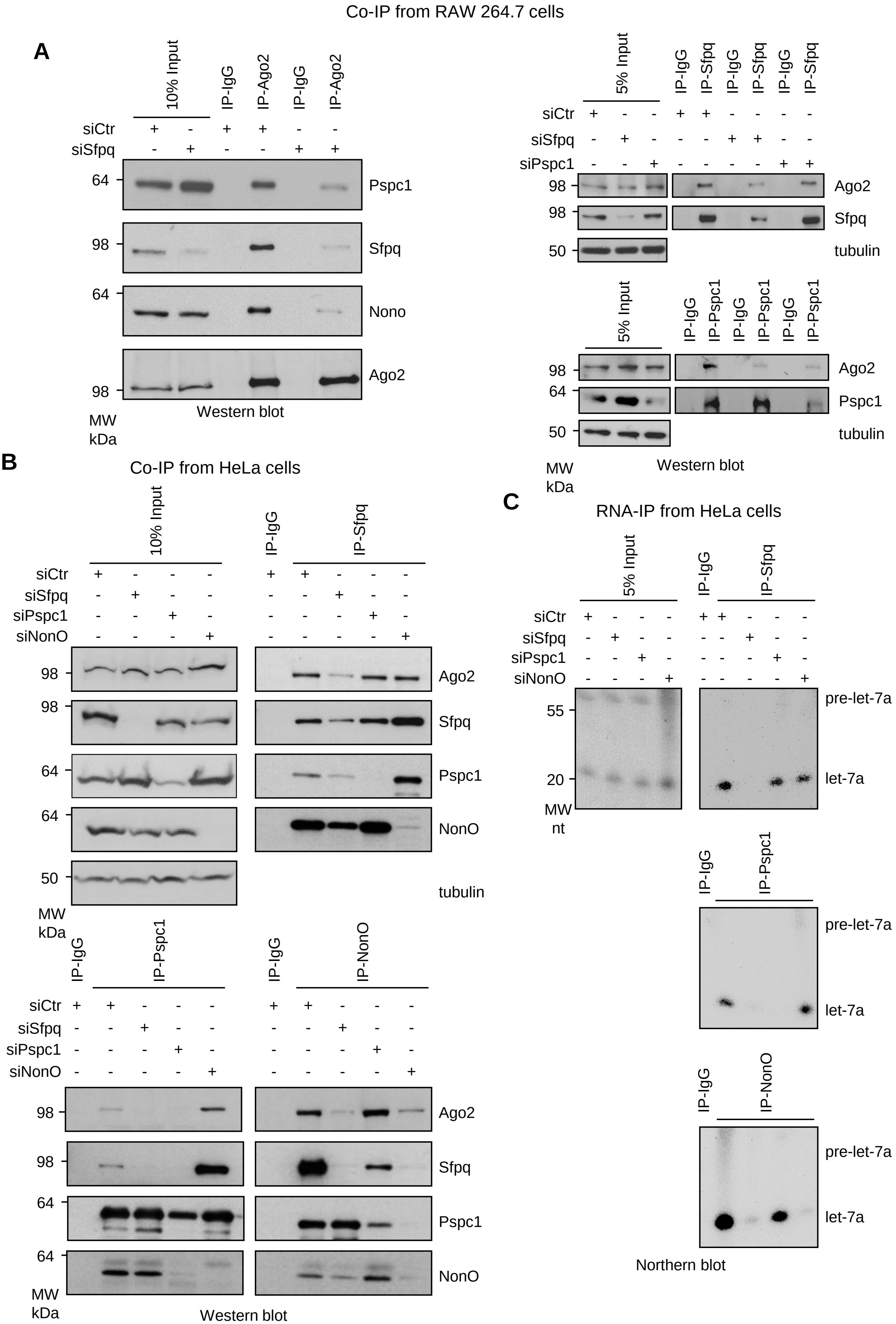

Co-IP from RAW 264.7 cells
A
10% Input
IP-IgG
IP-Ago2
IP-IgG
IP-Ago2
siCtr
siSfpq
64
98
64
98
Pspc1
Sfpq
Nono
Ago2
MW kDa
Western blot
5% Input
IP-IgG
IP-Sfpq
siPspc1
50
Ago2
Sfpq
tubulin
IP-Pspc1
Pspc1
B
Co-IP from HeLa cells
siNonO
NonO
IP-NonO
C
RNA-IP from HeLa cells
55
20
MW nt
pre-let-7a
let-7a
Northern blot

**Supplementary Figure 3. Sfpq mediates the interaction between the miRISC and Pspc1 or NonO.** Co-IP of the indicated endogenous proteins from (A) RAW 264.7 or (B) HeLa cells. Cells were transfected with the indicated siRNAs. Tubulin served as control for the knockdown. (C) RNA-IP of let-7a with the endogenous Sfpq, Pspc1, or NonO. HeLa cells were transfected with the indicated siRNAs. Cell extracts were immunoprecipitated with the indicated antibodies, RNA was purified and analyzed by Northern blotting.

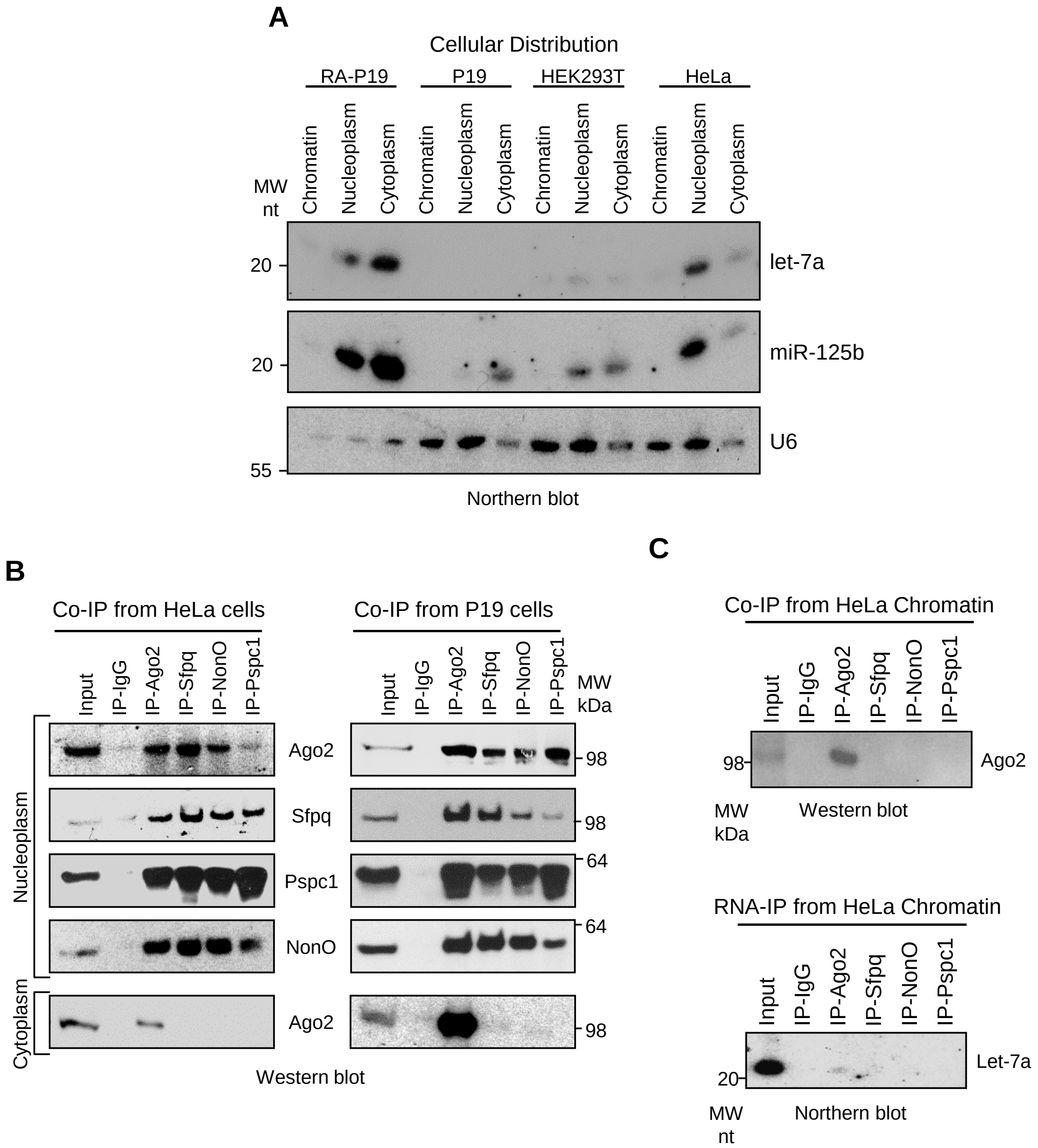
A
Cellular Distribution
RA-P19
P19
HEK293T
HeLa
Chromatin
Nucleoplasm
Cytoplasm
MW nt
20
20
55
let-7a
miR-125b
U6
Northern blot
B
Co-IP from HeLa cells
Co-IP from P19 cells
Input
IP-IgG
IP-Ago2
IP-Sfpq
IP-NonO
IP-Pspc1
MW kDa
Nucleoplasm
Cytoplasm
Ago2
Sfpq
Pspc1
NonO
Ago2
98
98
64
64
98
Western blot
C
Co-IP from HeLa Chromatin
Input
IP-IgG
IP-Ago2
IP-Sfpq
IP-NonO
IP-Pspc1
98
Ago2
MW kDa
Western blot
RNA-IP from HeLa Chromatin
Input
IP-IgG
IP-Ago2
IP-Sfpq
IP-NonO
IP-Pspc1
20
Let-7a
MW nt
Northern blot

**Supplementary Figure 4. Sfpq, Pspc1 and NonO interact with miRISC in the nucleoplasm.** (A) Northern blot analysis of chromatin, nucleoplasm, or cytoplasm from HeLa, HEK293T, P19, or retinoic acid (RA)-treated P19 cells using the indicated probes. (B) Co-IP of the indicated endogenous proteins and the indicated proteins from nucleoplasmic or cytoplasmic extracts of HeLa (left panel) or P19 cells (right panel). (C) Co-IP (upper panel) and RNA-IP (lower panel) of the indicated endogenous proteins and let-7a from chromatin extracts of HeLa cells.

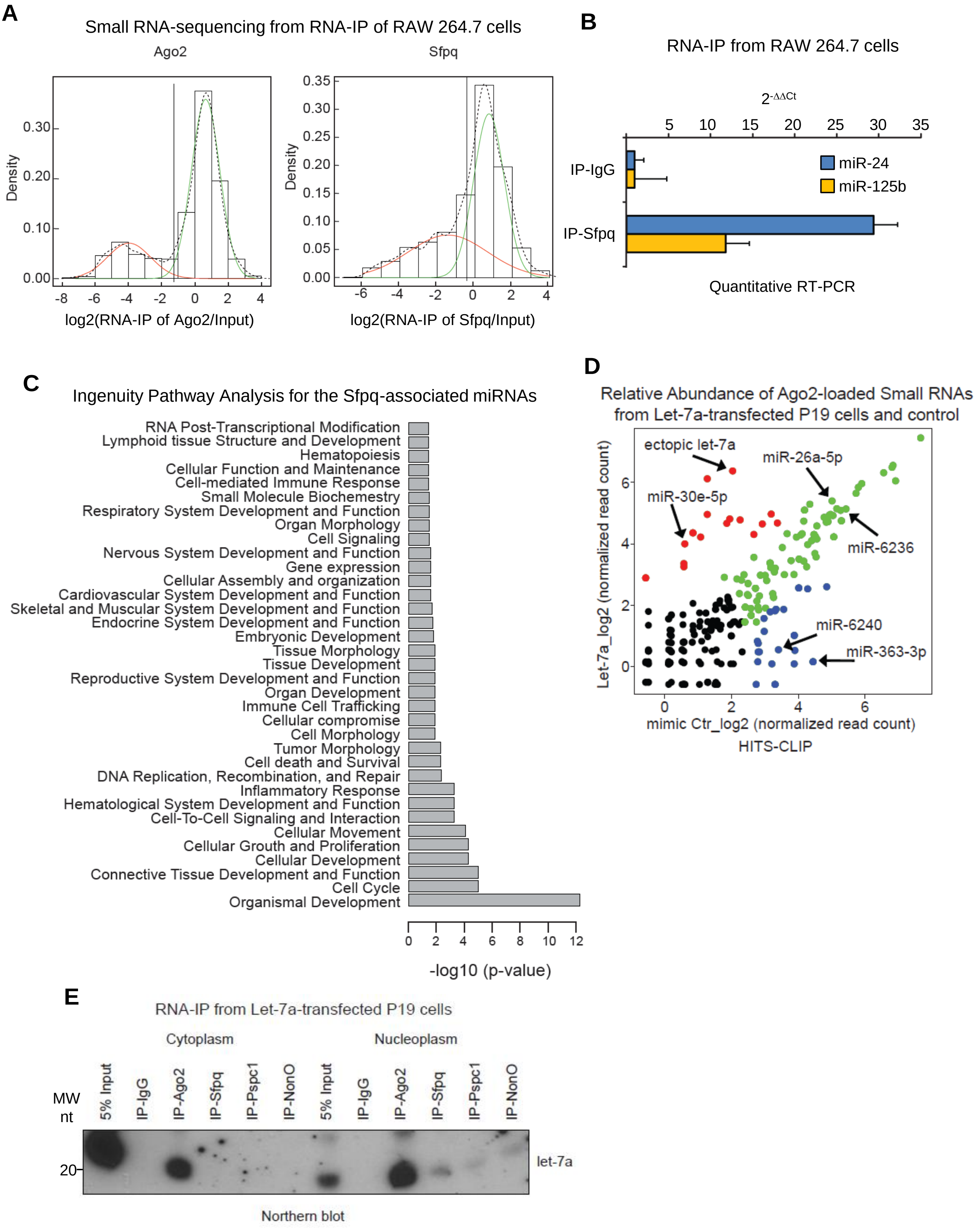

A
Small RNA-sequencing from RNA-IP of RAW 264.7 cells
Ago2
Sfpq
Density
log2(RNA-IP of Ago2/Input)
log2(RNA-IP of Sfpq/Input)
B
RNA-IP from RAW 264.7 cells
2-ΔΔCt
IP-IgG
IP-Sfpq
miR-24
miR-125b
Quantitative RT-PCR
C
Ingenuity Pathway Analysis for the Sfpq-associated miRNAs
RNA Post-Transcriptional Modification
Lymphoid tissue Structure and Development
Hematopoiesis
Cellular Function and Maintenance
Cell-mediated Immune Response
Small Molecule Biochemestry
Respiratory System Development and Function
Organ Morphology
Cell Signaling
Nervous System Development and Function
Gene expression
Cellular Assembly and organization
Cardiovascular System Development and Function
Skeletal and Muscular System Development and Function
Endocrine System Development and Function
Embryonic Development
Tissue Morphology
Tissue Development
Reproductive System Development and Function
Organ Development
Immune Cell Trafficking
Cellular compromise
Cell Morphology
Tumor Morphology
Cell death and Survival
DNA Replication, Recombination, and Repair
Inflammatory Response
Hematological System Development and Function
Cell-To-Cell Signaling and Interaction
Cellular Movement
Cellular Grouth and Proliferation
Cellular Development
Connective Tissue Development and Function
Cell Cycle
Organismal Development
-log10 (p-value)
D
Relative Abundance of Ago2-loaded Small RNAs
from Let-7a-transfected P19 cells and control
ectopic let-7a
miR-26a-5p
miR-30e-5p
miR-6236
miR-6240
miR-363-3p
Let-7a_log2 (normalized read count)
mimic Ctr_log2 (normalized read count)
HITS-CLIP
E
RNA-IP from Let-7a-transfected P19 cells
Cytoplasm
Nucleoplasm
MW
nt
5% Input
IP-IgG
IP-Ago2
IP-Sfpq
IP-Pspc1
IP-NonO
20
let-7a
Northern blot

**Supplementary Figure 5. Sfpq associates to miRNAs.** (A) Small RNA log2(RNA-IP Input^-1) distribution (dotted line) from small RNA sequencing of Ago2 (left panel) or Sfpq (right panel) RNA-IP from RAW 264.7 cells. Main distributions are composed by two Gaussians: the green line represents small RNA mostly expressed in the RNA-IPs whereas the red line represents the small RNAs mostly expressed in the inputs. A LOD approach was set up to find a threshold to discriminate the two populations (vertical solid line). (B) RNA-IP of Sfpq and miR-24 or miR-125b in RAW 264.7 cells. Total cell extracts were immunoprecipitated and RNA in the immunocomplexes was analyzed by quantitative RT-PCR. Data are normalized with U2 snRNA and presented as the mean ± s.e.m. (n = 4). (C) P-values of the Ingenuity Pathway Analysis for the Sfpq-associated miRNAs. (D) Relative abundance of small RNAs found in Ago2 HITS-CLIP experiments in let-7a-transfected P19 cells and control. Red: small RNAs enriched in let-7a condition. Blue: small RNAs enriched in mimic control condition (Ctr). Green: small RNAs found in equal levels in both conditions. Black: not significantly enriched small RNAs. (E) RNA-IP of let-7a with Ago2, Sfpq, Pspc1, or NonO in let-7a-transfected P19 cells. Cell extracts were immunoprecipitated from fractionated nucleoplasm or cytoplasm with the indicated antibodies, RNA was purified and analyzed by Northern blotting.

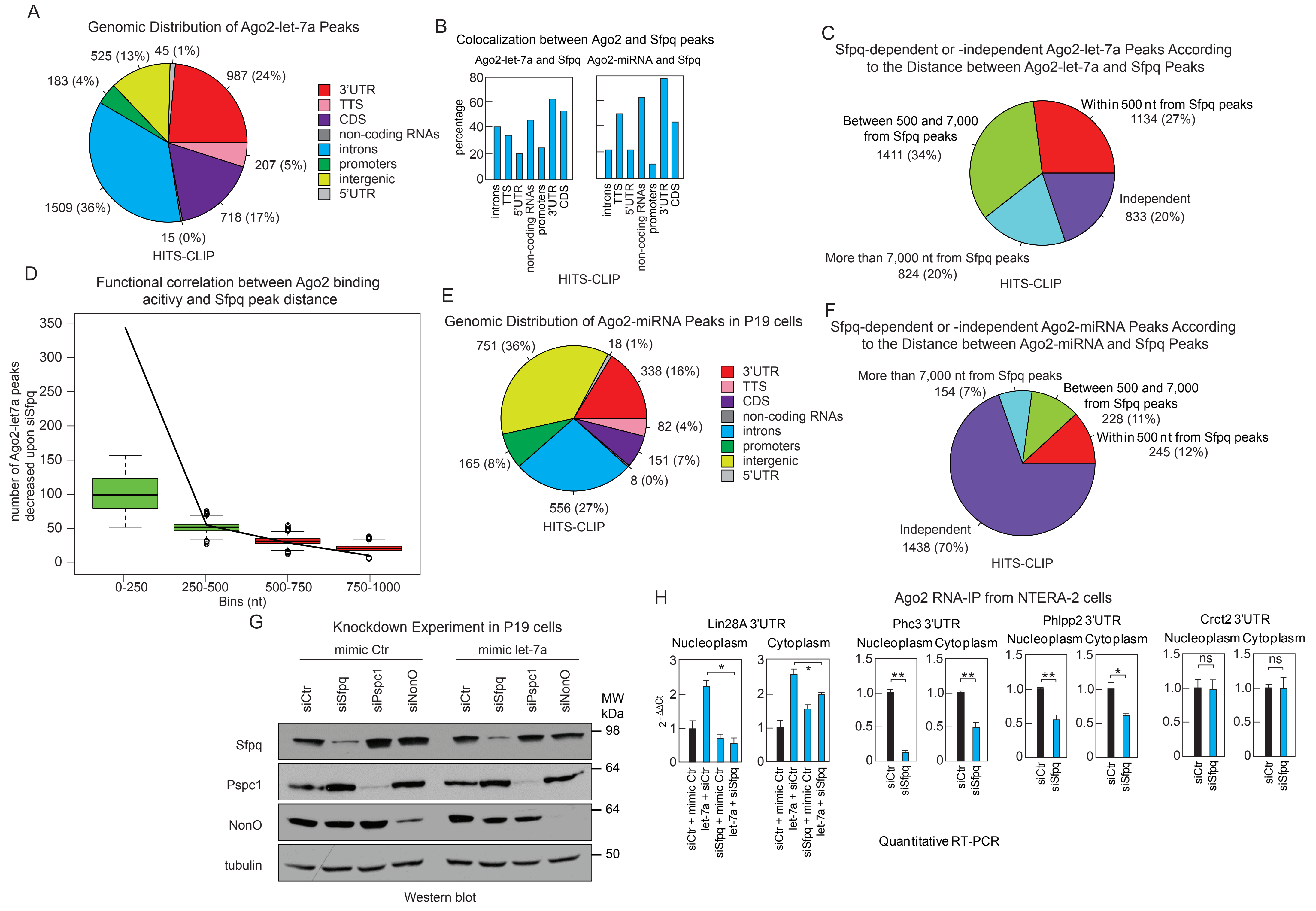

A
Genomic Distribution of Ago2-let-7a Peaks
3'UTR
TTS
CDS
non-coding RNAs
introns
promoters
intergenic
5'UTR
HITS-CLIP
B
Colocalization between Ago2 and Sfpq peaks
Ago2-let-7a and Sfpq
Ago2-miRNA and Sfpq
percentage
HITS-CLIP
C
Sfpq-dependent or -independent Ago2-let-7a Peaks According to the Distance between Ago2-let-7a and Sfpq Peaks
Within 500 nt from Sfpq peaks 1134 (27%)
Between 500 and 7,000 from Sfpq peaks 1411 (34%)
Independent 833 (20%)
More than 7,000 nt from Sfpq peaks 824 (20%)
HITS-CLIP
D
Functional correlation between Ago2 binding acitivy and Sfpq peak distance
number of Ago2-let7a peaks decreased upon siSfpq
Bins (nt)
E
Genomic Distribution of Ago2-miRNA Peaks in P19 cells
HITS-CLIP
F
Sfpq-dependent or -independent Ago2-miRNA Peaks According to the Distance between Ago2-miRNA and Sfpq Peaks
More than 7,000 nt from Sfpq peaks 154 (7%)
Between 500 and 7,000 from Sfpq peaks 228 (11%)
Within 500 nt from Sfpq peaks 245 (12%)
Independent 1438 (70%)
HITS-CLIP
G
Knockdown Experiment in P19 cells
mimic Ctr
mimic let-7a
siCtr
siSfpq
siPspc1
siNonO
MW kDa
Sfpq
Pspc1
NonO
tubulin
Western blot
H
Ago2 RNA-IP from NTERA-2 cells
Lin28A 3'UTR
Phc3 3'UTR
Phlpp2 3'UTR
Crct2 3'UTR
Nucleoplasm
Cytoplasm
siCtr + mimic Ctr
let-7a + siCtr
siSfpq + mimic Ctr
let-7a + siSfpq
Quantitative RT-PCR

**Supplementary Figure 6. Global analysis of Sfpq-dependent miRNA targeting by HITS-CLIP analysis in P19 cells.** (A) Ago2-let-7a peaks from dCLIP analysis of HITS-CLIP data grouped in different genomic regions. HITS-CLIP experiments were performed in let-7a-transfected P19 cells and mimic control. (B) Genomic distribution of Ago2-let-7a or Ago2-miRNA peaks that colocalize with Sfpq peaks identified by HITS-CLIP analysis. (C) Percentage of the Sfpq-dependent or independent Ago2-let-7a peaks. The Sfpq-dependent Ago2-let-7a peaks were further grouped considering their position to the closest Sfpq peak determined by HITS-CLIP analysis in P19 cells. The groups considered are the following: *(i)* close distance to Sfpq peaks (less than 500 nt); *(ii)* far distance to Sfpq peaks (between 500 and 7,000 nt); and *(iii)* very far distance (more than 7,000 nt). (D) Number of reduced Ago2-let-7a peaks upon Sfpq knockdown in the 3'UTR divided in bins of 250 nt from Sfpq Peaks (black solid line). In the box-plots are represented the 10,000 times shuffling of the distance between Sfpq and the reduced Ago2-let-7a peaks upon Sfpq knockdown in each bin. The colors in the plot indicate whether the Z score was positive (green) or negative (red). (E) Genomic distribution of endogenously expressed miRNA binding sites (Ago2-miRNA peaks) according to Bottini et al.[2]. (F) Genomic distribution of the Sfpq-dependent or independent endogenous Ago2-miRNA peaks. The Sfpq-dependent Ago2-miRNA peaks were further grouped considering their position to the closest Sfpq peak determined by HITS-CLIP analysis in P19 cells. (G) Immunoblot showing the effectiveness and the specificity of the knockdown for Sfpq, Pspc1, and NonO in P19 cells transfected with mimic let-7a or mimic control. (H) NTERA-2 cells were transfected with the indicated molecules before lysate them by fractionating nucleoplasm and cytoplasm. Before Ago2 IP the lysates were partially digested with 10μg ml^-1 RNase A for 30 min at room temperature. RNA was purified from the immunocomplexes and from 5% of input, and analyzed by RT-qPCR using oligonucleotide probes surrounding let-7a binding site for Lin28A 3'UTR and the miR-302b binding sites for Phc3, Phlpp2 or Crct2 3'UTRs, identified by HITS-CLIP analysis. Data are normalized with their own inputs and presented as the mean ± s.e.m. (n = 3). Student's t-test for Phc3, Phlpp2 or Crct2 3'UTRs and one-way ANOVA followed by Tukey's *post hoc* test for Lin28A 3'UTR: *P<0.05, **P<0.01, ns (not significant).

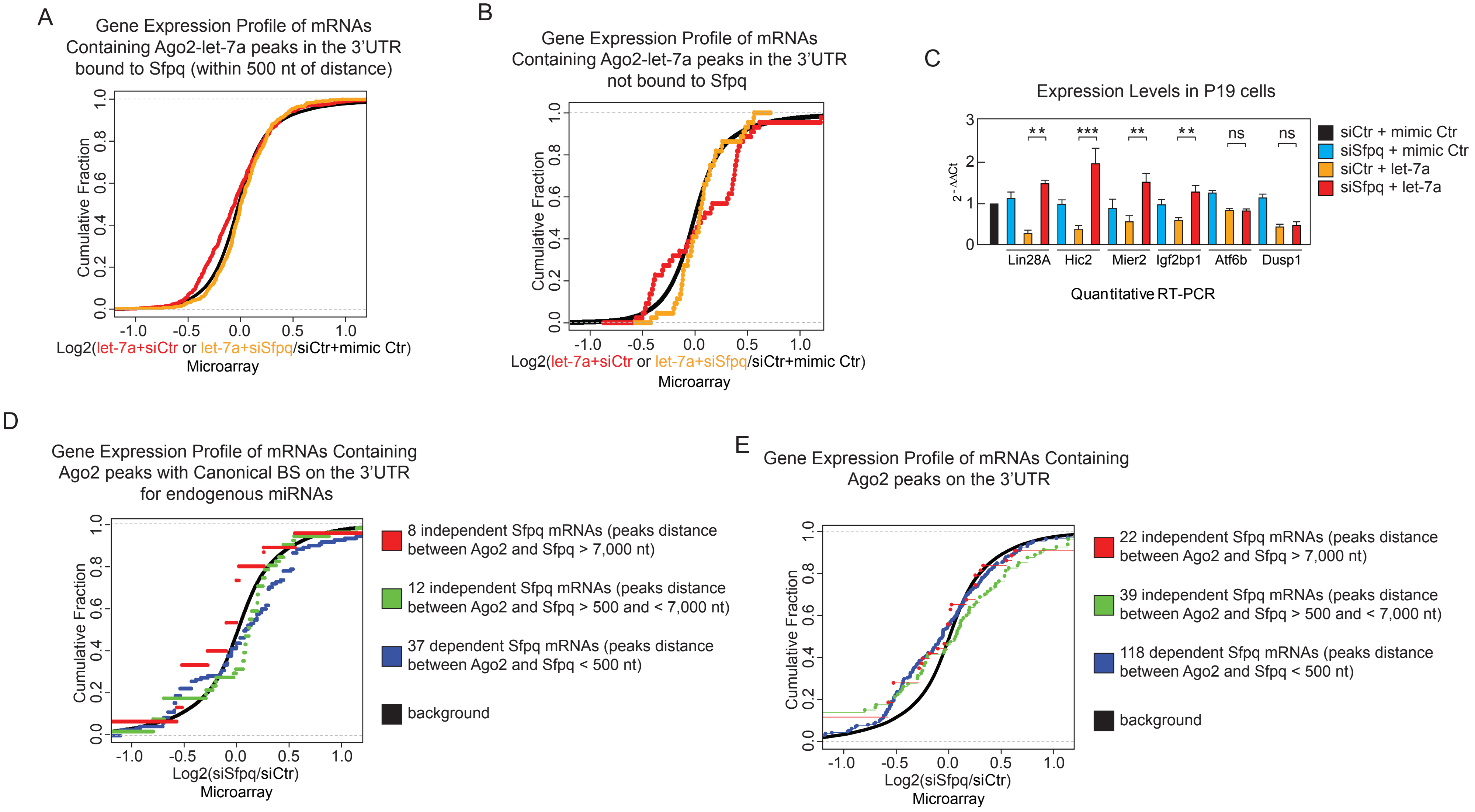
A
Gene Expression Profile of mRNAs Containing Ago2-let-7a peaks in the 3'UTR bound to Sfpq (within 500 nt of distance)
Cumulative Fraction
0.0
0.2
0.4
0.6
0.8
1.0
-1.0
-0.5
0.0
0.5
1.0
Log2(let-7a+siCtr or let-7a+siSfpq/siCtr+mimic Ctr)
Microarray
B
Gene Expression Profile of mRNAs Containing Ago2-let-7a peaks in the 3'UTR not bound to Sfpq
Cumulative Fraction
Log2(let-7a+siCtr or let-7a+siSfpq/siCtr+mimic Ctr)
Microarray
C
Expression Levels in P19 cells
2^-ΔΔCt
0
1
2
3
**
***
**
**
ns
ns
Lin28A
Hic2
Mier2
Igf2bp1
Atf6b
Dusp1
Quantitative RT-PCR
siCtr + mimic Ctr
siSfpq + mimic Ctr
siCtr + let-7a
siSfpq + let-7a
D
Gene Expression Profile of mRNAs Containing Ago2 peaks with Canonical BS on the 3'UTR for endogenous miRNAs
Cumulative Fraction
Log2(siSfpq/siCtr)
Microarray
8 independent Sfpq mRNAs (peaks distance between Ago2 and Sfpq > 7,000 nt)
12 independent Sfpq mRNAs (peaks distance between Ago2 and Sfpq > 500 and < 7,000 nt)
37 dependent Sfpq mRNAs (peaks distance between Ago2 and Sfpq < 500 nt)
background
E
Gene Expression Profile of mRNAs Containing Ago2 peaks on the 3'UTR
Cumulative Fraction
Log2(siSfpq/siCtr)
Microarray
22 independent Sfpq mRNAs (peaks distance between Ago2 and Sfpq > 7,000 nt)
39 independent Sfpq mRNAs (peaks distance between Ago2 and Sfpq > 500 and < 7,000 nt)
118 dependent Sfpq mRNAs (peaks distance between Ago2 and Sfpq < 500 nt)
background

**Supplementary Figure 7. Sfpq promotes post-transcriptional silencing mediated by miRNAs.** Gene expression differences in let-7a-tranfected P19 cells upon Sfpq knockdown and control. Expression is plotted for mRNAs containing Ago2-let-7a peaks in the 3'UTR localized in a (A) closely distance to Sfpq peaks (less than 500 nt) or (B) very far distance-unbound to Sfpq peaks (more than 7,000 nt), and upon Sfpq knockdown. (C) Sfpq knockdown inhibits the let-7a-dependent downregulation of selected direct target mRNAs. mRNA levels were estimated by RT-qPCR in let-7a-transfected P19 cells. Data are normalized with U2 snRNA and presented as the mean ± s.e.m. (n = 6). Gene expression differences in siSfpq-transfected P19 cells and control. Differential expression is plotted for mRNAs containing Ago2 peaks in the 3'UTR (D) bearing only the canonical binding sites (BS) for the 20 most expressed miRNAs or (E) the all set. One-way ANOVA followed by Tukey's *post hoc* test for C: $^{**}P < 0.01$, $^{***}P < 0.001$, ns (not significant).

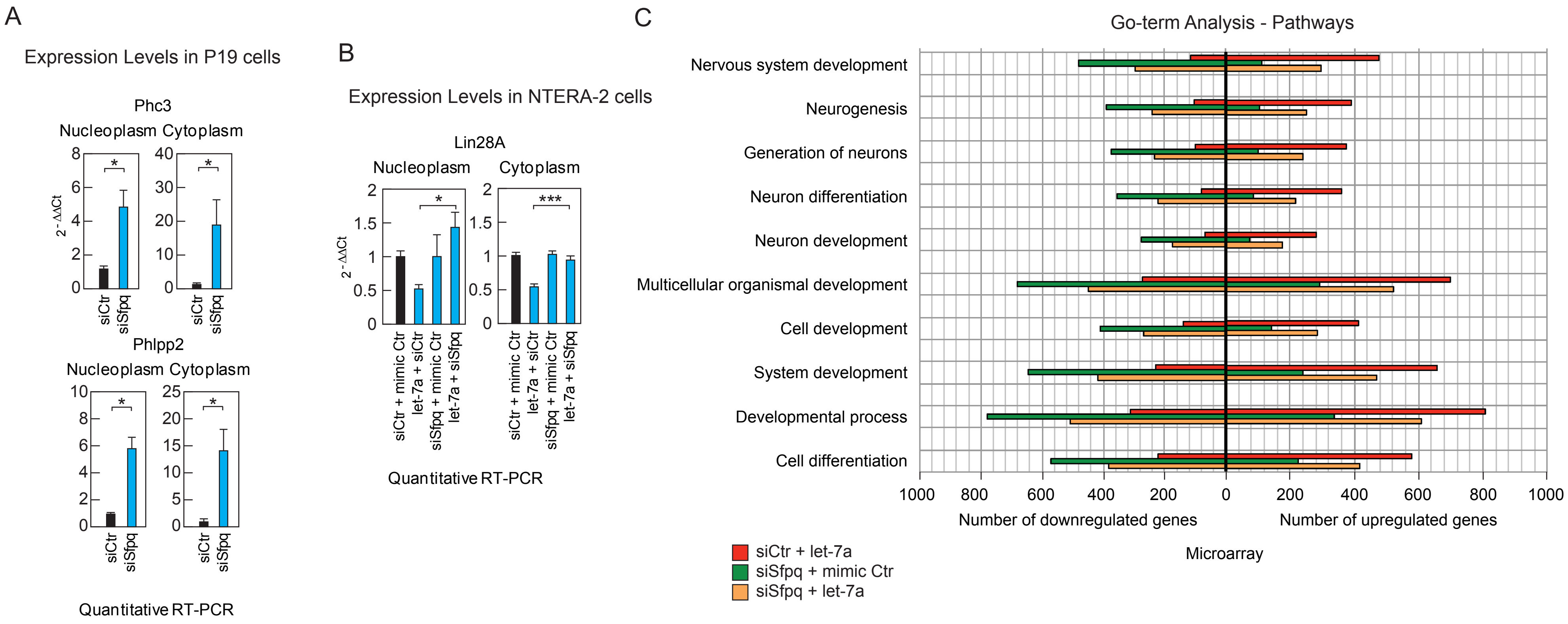
A
Expression Levels in P19 cells
Phc3
Nucleoplasm Cytoplasm
2^-ΔΔCt
siCtr
siSfpq
Phlpp2
Nucleoplasm Cytoplasm
Quantitative RT-PCR
B
Expression Levels in NTERA-2 cells
Lin28A
Nucleoplasm
Cytoplasm
2^-ΔΔCt
siCtr + mimic Ctr
let-7a + siCtr
siSfpq + mimic Ctr
let-7a + siSfpq
Quantitative RT-PCR
C
Go-term Analysis - Pathways
Nervous system development
Neurogenesis
Generation of neurons
Neuron differentiation
Neuron development
Multicellular organismal development
Cell development
System development
Developmental process
Cell differentiation
Number of downregulated genes
Number of upregulated genes
siCtr + let-7a
siSfpq + mimic Ctr
siSfpq + let-7a
Microarray

**Supplementary Figure 8. Sfpq promotes post-transcriptional silencing mediated by miRNAs in both nucleoplasm and cytoplasm to regulate gene expression programs.** (A) P19 or (B) NTERA-2 cells were transfected with the indicated molecules before lysate them by fractionating nucleoplasm and cytoplasm to measure the indicated miR-302b-target mRNAs and the let-7a-target Lin28A mRNA, respectively. RNA was purified and analyzed by RT-qPCR. Data are normalized with U2 snRNA and presented as the mean ± s.e.m. (n = 3). (C) Number of downregulated or upregulated transcripts in the top 10 enriched pathways from GO Term analysis from microarray data of the indicated conditions in P19 cells. Student's t-test for A and one-way ANOVA followed by Tukey's *post hoc* test for B: *P < 0.05, **P < 0.01, ns (not significant).

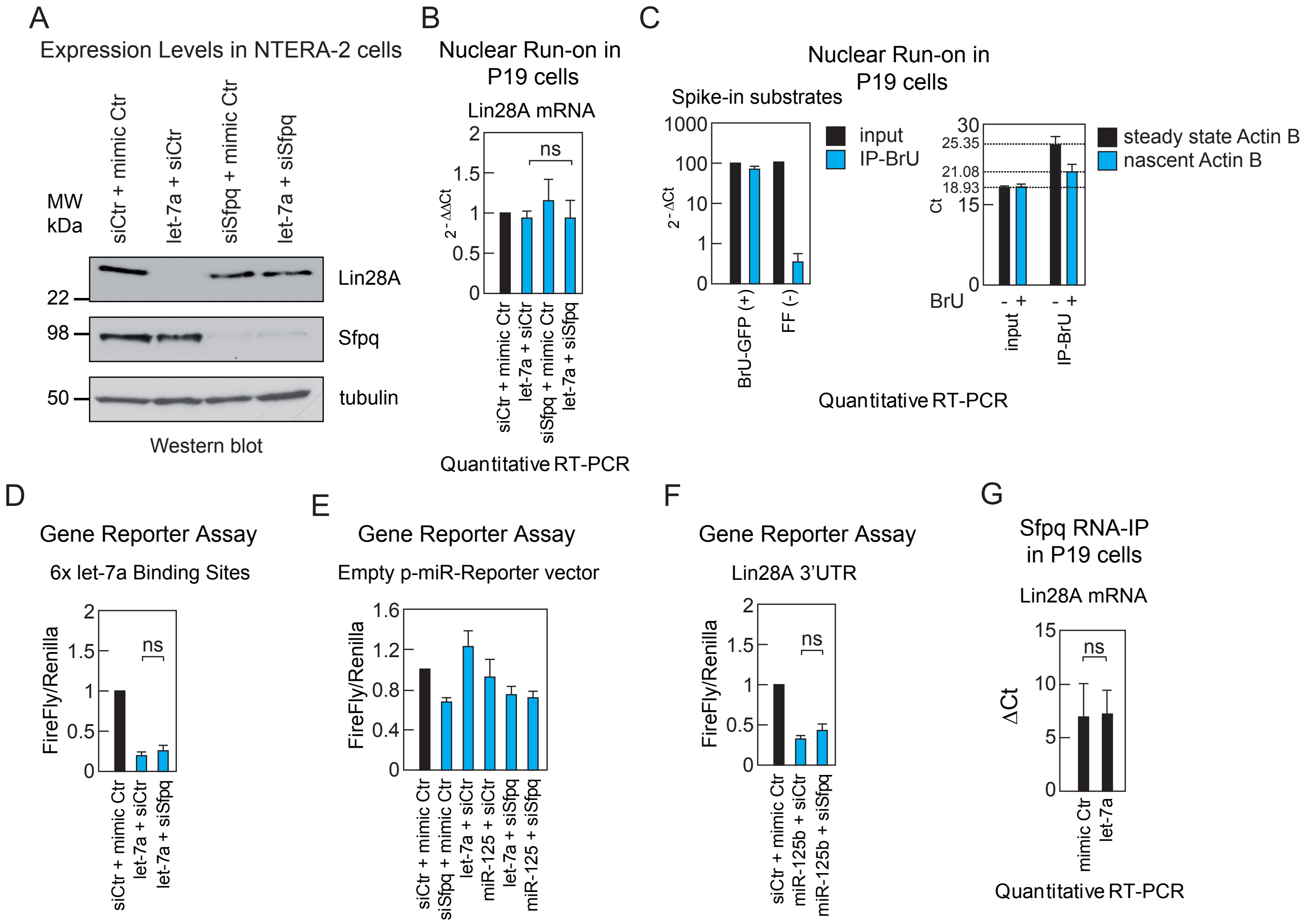
A
Expression Levels in NTERA-2 cells
siCtr + mimic Ctr
let-7a + siCtr
siSfpq + mimic Ctr
let-7a + siSfpq
MW kDa
22
98
50
Lin28A
Sfpq
tubulin
Western blot
B
Nuclear Run-on in P19 cells
Lin28A mRNA
ns
2-ΔΔCt
siCtr + mimic Ctr
let-7a + siCtr
siSfpq + mimic Ctr
let-7a + siSfpq
Quantitative RT-PCR
C
Nuclear Run-on in P19 cells
Spike-in substrates
input
IP-BrU
2-ΔCt
BrU-GFP (+)
FF (-)
steady state Actin B
nascent Actin B
25.35
21.08
18.93
Ct
BrU
input
IP-BrU
Quantitative RT-PCR
D
Gene Reporter Assay
6x let-7a Binding Sites
FireFly/Renilla
ns
siCtr + mimic Ctr
let-7a + siCtr
let-7a + siSfpq
E
Gene Reporter Assay
Empty p-miR-Reporter vector
FireFly/Renilla
siCtr + mimic Ctr
siSfpq + mimic Ctr
let-7a + siCtr
miR-125 + siCtr
let-7a + siSfpq
miR-125 + siSfpq
F
Gene Reporter Assay
Lin28A 3'UTR
FireFly/Renilla
ns
siCtr + mimic Ctr
miR-125b + siCtr
miR-125b + siSfpq
G
Sfpq RNA-IP in P19 cells
Lin28A mRNA
ΔCt
ns
mimic Ctr
let-7a
Quantitative RT-PCR

**Supplementary Figure 9. Sfpq promotes post-transcriptional silencing of Lin28A mRNA mediated by let-7a.** (A) Immunoblot analysis of Lin28A, Sfpq, and tubulin in NTERA-2 cells transfected with mimic let-7a and or siSfpq. RT-qPCR analysis of the (B) nascent Lin28A mRNA, (C left panel) spike-in negative (Fire Fly or FF) and positive (GFP) RNA controls, or (C right panel) actin B as endogenously expressed control. Data are presented as the mean ± s.e.m. (n = 3), and normalized with actin B for Lin28A mRNA, or with the input for both spike-in RNAs. (D) Relative luciferase activity of reporter constructs containing only the sequence of 6 canonical binding sites for let-7a from mouse Lin28a 3'UTR in HEK293T cells transfected with mimic let-7a or siSfpq, as indicated. The data were normalized using Renilla activity. Relative luciferase activity of (E) reporter empty vector sequence or (F) the construct containing the mouse Lin28a 3'UTR in HEK293T cells transfected with the indicated molecules. (G) RNA-IP of Sfpq and Lin28A mRNA in let-7a-transfected P19 cells and control. Total cell extracts were immunoprecipitated and RNA in the immunocomplexes was analyzed by quantitative RT-PCR. Data are normalized with the inputs and presented as the mean ± s.e.m. (n = 4). Student's t-test for G and one-way ANOVA followed by Tukey's *post hoc* test for B, D and F: ns (not significant).

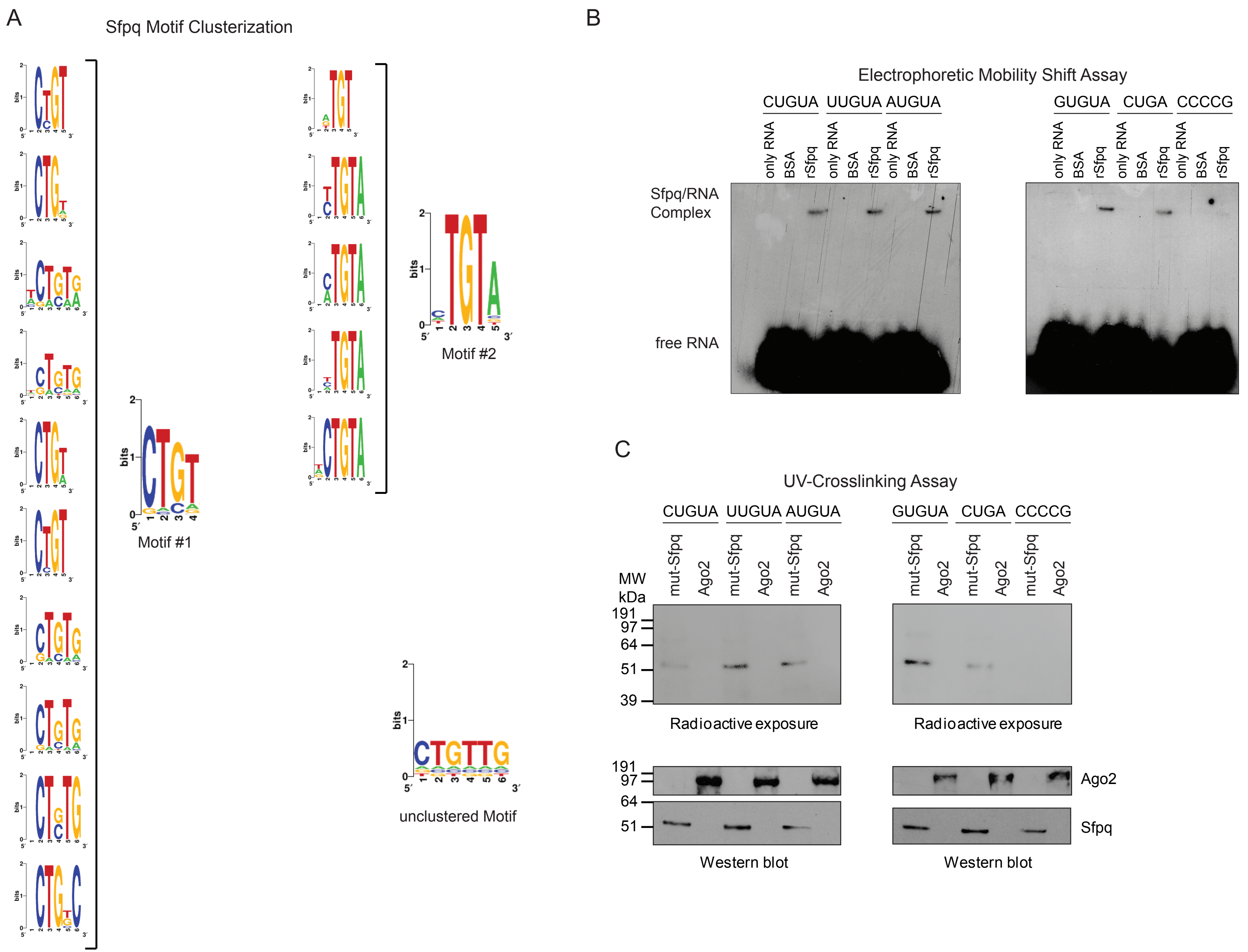

A
Sfpq Motif Clusterization
Motif #1
Motif #2
unclustered Motif
B
Electrophoretic Mobility Shift Assay
CUGUA UUGUA AUGUA
GUGUA CUGA CCCCG
only RNA BSA rSfpq
Sfpq/RNA Complex
free RNA
C
UV-Crosslinking Assay
CUGUA UUGUA AUGUA
GUGUA CUGA CCCCG
mut-Sfpq Ago2
MW kDa
191
97
64
51
39
Radioactive exposure
Ago2
Sfpq
Western blot

**Supplementary Figure 10. Search and validation of Sfpq binding motif(s).** (A) Output results of the Sfpq motif clusterization with the program Gimmemotifs. (B) EMSA of the indicated Sfpq-binding RNA sequence and the full length of human recombinant wild-type Sfpq (100 nM). Recombinant protein and $^{32}$P-labeled RNA substrates were incubated at room temperature for 30 min and resolved on 10% polyacrylamide native gel. (C) UV-crosslinking assay to analyze the interaction of the recombinant Sfpq-214–598 quadruple mutant (L535A, L539A, L546A, and M549A) (100 nM) or the full length of human recombinant Ago2 (100 nM) with the indicated $^{32}$P-labeled RNA oligonucleotides containing two copies of the indicated Sfpq-binding RNA sequence or negative control sequence. After radioactive exposure the membrane were subject of immunoblot analyses with the indicated antibodies.

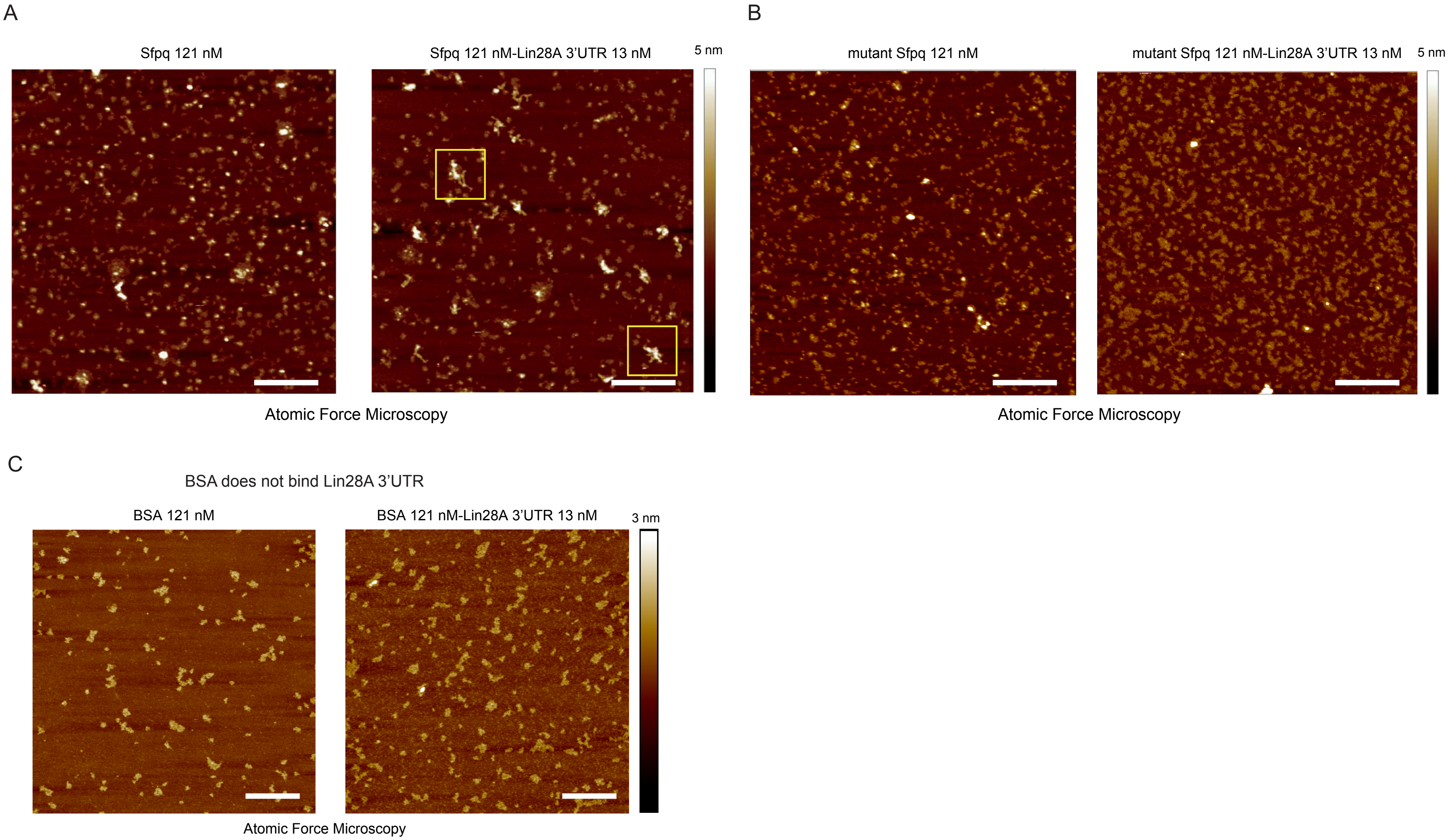
A
Sfpq 121 nM
Sfpq 121 nM-Lin28A 3’UTR 13 nM
5 nm
Atomic Force Microscopy
B
mutant Sfpq 121 nM
mutant Sfpq 121 nM-Lin28A 3’UTR 13 nM
5 nm
Atomic Force Microscopy
C
BSA does not bind Lin28A 3’UTR
BSA 121 nM
BSA 121 nM-Lin28A 3’UTR 13 nM
3 nm
Atomic Force Microscopy

**Supplementary Figure 11. Sfpq aggregates onto Lin28A 3'UTR.** Top view of topographic Atomic Force Microscope (AFM) images of (A) wild-type Sfpq protein (left panel) and wild-type Sfpq-Lin28A 3'UTR RNA complexes (right panel - in yellow squares some complexes are shown) or (B) Sfpq-214–598 quadruple mutant (L535A, L539A, L546A, and M549A) protein (left panel) and mutant Sfpq-Lin28A 3'UTR RNA complexes (right panel) adsorbed on untreated mica surface. The color bar on the right of each image represents the height scale with a maximum of 5nm. Scale bar corresponds to 200 nm. (C) Top view of topographic AFM images of BSA (left panel) and BSA previously incubated with Lin28 3'UTR (right panel). The color bar on the right represents the height scale with a maximum of 3nm. Scale bar corresponds to 200 nm.

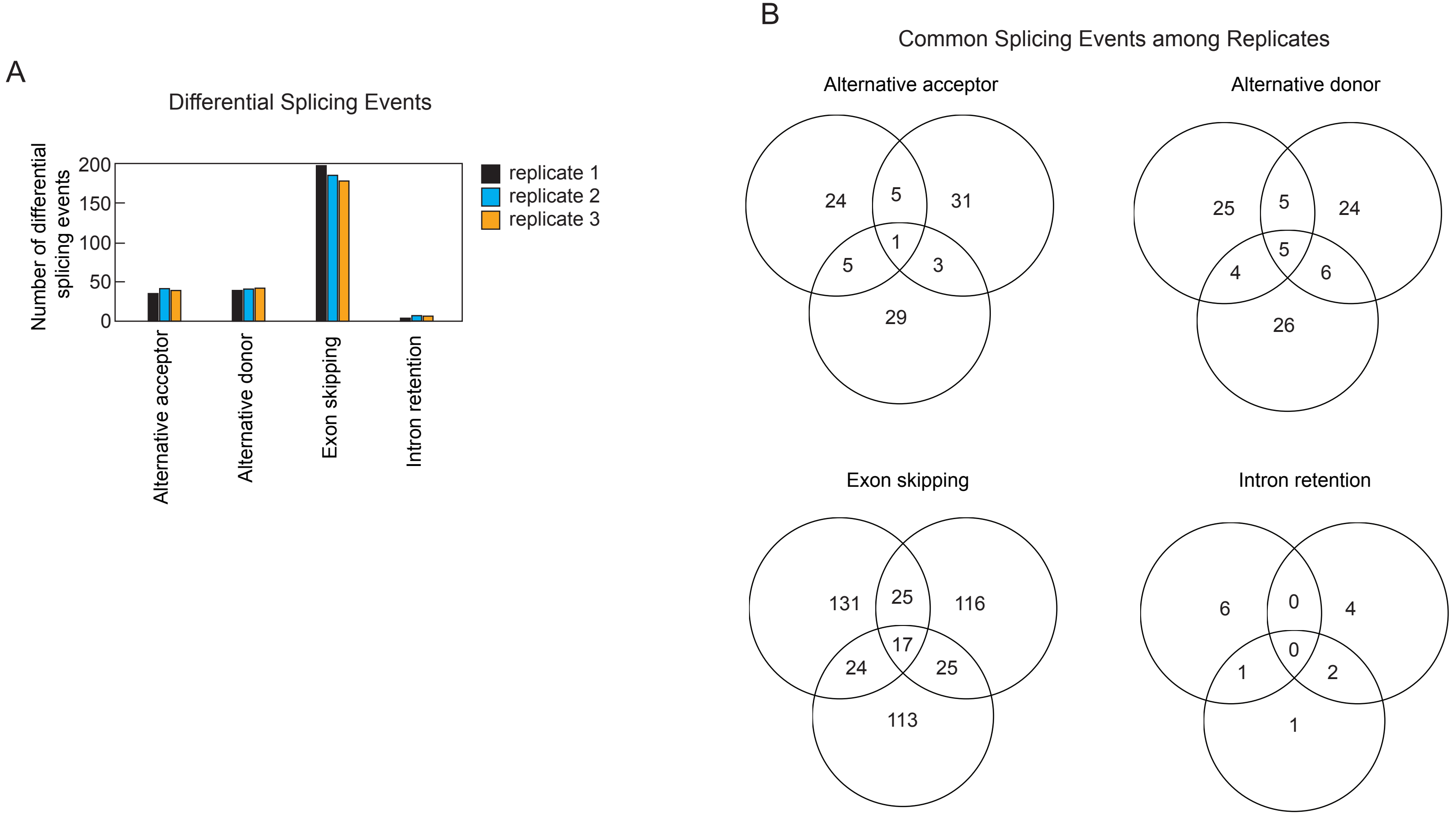
A
Differential Splicing Events
Number of differential splicing events
200
150
100
50
0
replicate 1
replicate 2
replicate 3
Alternative acceptor
Alternative donor
Exon skipping
Intron retention
B
Common Splicing Events among Replicates
Alternative acceptor
24
5
31
1
5
3
29
Alternative donor
25
5
24
5
4
6
26
Exon skipping
131
25
116
17
24
25
113
Intron retention
6
0
4
0
1
2
1

**Supplementary Figure 12. Sfpq does not control alternative splicing on the same set of 3'UTRs on which it controls miRNA targeting.** (A) Histogram shows the number of differentially splicing events obtained by "SpliceTrap" program comparing RNA-sequencing samples of siSfpq-transfected P19 cells to siCtr-transfected P19 cells from each replicates, by RNA-sequencing analysis. (B) Venn diagrams show the number of differentially splicing events in common between the three replicates for each event category.

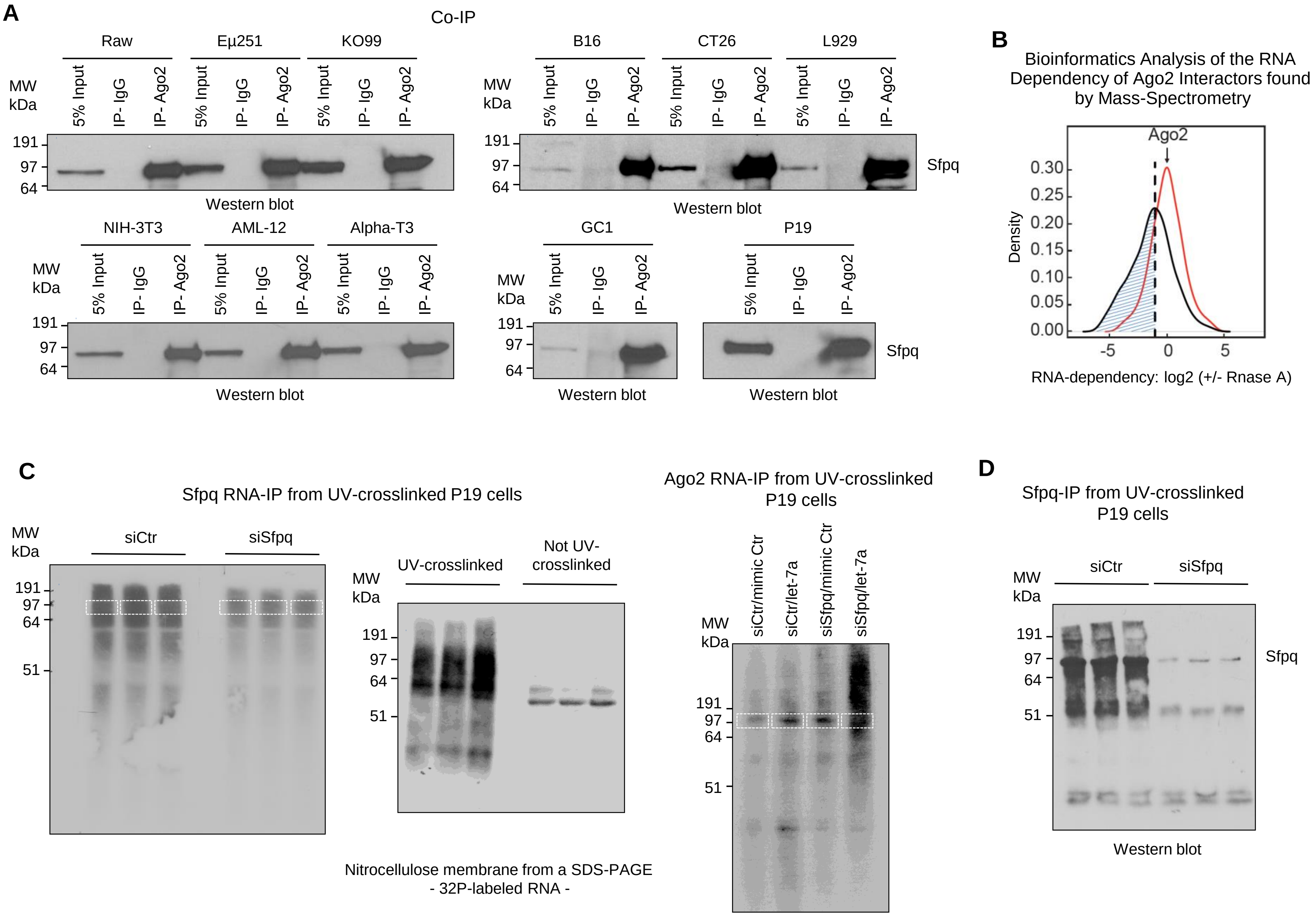
A
Co-IP
Raw
Eμ251
KO99
B16
CT26
L929
MW kDa
5% Input
IP- IgG
IP- Ago2
191
97
64
Sfpq
Western blot
NIH-3T3
AML-12
Alpha-T3
GC1
P19
B
Bioinformatics Analysis of the RNA Dependency of Ago2 Interactors found by Mass-Spectrometry
Ago2
Density
0.30
0.25
0.20
0.15
0.10
0.05
0.00
-5
0
5
RNA-dependency: log2 (+/- Rnase A)
C
Sfpq RNA-IP from UV-crosslinked P19 cells
siCtr
siSfpq
51
UV-crosslinked
Not UV-crosslinked
Ago2 RNA-IP from UV-crosslinked P19 cells
siCtr/mimic Ctr
siCtr/let-7a
siSfpq/mimic Ctr
siSfpq/let-7a
Nitrocellulose membrane from a SDS-PAGE
- 32P-labeled RNA -
D
Sfpq-IP from UV-crosslinked P19 cells
Western blot

**Supplementary Figure 13.** (A) Sfpq and Ago2 interaction is conserved in various mouse cell types. Co-IP of endogenous Sfpq and Ago2 in the indicated cell lines. RAW 264.7 cells are macrophages; Eμ251 and KO99 cells are lymphocytes; B16 cells are melanocytes; CT26 cells are colon carcinoma cells; L929 and NIH-3T3 cells are fibroblasts; AML-12 cells are hepatocytes; alpha-T3 are from pituitary; GC1 are germ cells; P19 cells are embryonic stem cells. (B) Bioinformatics analysis about the RNA-dependency of Ago2 interactors from the MS analysis. Density plot of the log base 2 (-/+) RNase ratios (abundance scores) from all the data (shown in black) and the null distribution (shown in red). The vertical dashed black line indicates the 10% LOD cutoff that was used to classify a protein as RNA-dependent interactor (on the left of the dashed line). See the data analysis section of methods for an explanation of how the null distribution was calculated. (C) IP of either Sfpq-RNA complex (left panel) or Ago2-RNA complex (right panel) labeled with $^{32}P$ from P19 cells. The protein-RNA complex in dashed box was purified and used to prepare libraries for HITS-CLIP analysis. Illustrative images are shown. As control experiment for Sfpq HITS-CLIP experiment, we ran a protein gel of either UV-crosslinked or not UV-crosslinked Sfpq immunoprecipitated samples labeled with $^{32}P$ from P19 cells (middle panel). (D) As a further control for Sfpq HITS-CLIP experiment, we also immunoprecipitated endogenous Sfpq from UV-crosslinked P19 cells upon Sfpq knockdown or control followed by Sfpq immunoblotting.

## Supplementary References

1. Supek F, Bosnjak M, Skunca N, Smuc T. REVIGO summarizes and visualizes long lists of gene ontology terms. *PLoS One* **6**, e21800 (2011).

2. Bottini S, *et al.* From benchmarking HITS-CLIP peak detection programs to a new method for identification of miRNA-binding sites from Ago2-CLIP data. *Nucleic Acids Res* **45**, e71 (2017).
