# Supplementary material for: Post-transcriptional gene silencing mediated by microRNAs is controlled by nucleoplasmic Sfpq
Source: Nat Commun. 2017 Oct 30;8:1189. doi: 10.1038/s41467-017-01126-x (PMC5662751; doi:10.1038/s41467-017-01126-x)
Supplement: Supplementary file 3 — Description of Additional Supplementary Files [file 41467_2017_1126_MOESM3_ESM.pdf]

Description of Additional Supplementary Files

File Name: Supplementary Data 1
Description: Proteomic analysis of RAW 264.7 cells immunoprecipitated with anti-Ago2 antibody, IgG, or just protein G coupled with dynabeads on three biological replicates. Cell extracts were either digested with RNase A 10 mg ml-1 for 30 min or untreated before immunoprecipitation.

File Name: Supplementary Data 2
Description: Bioinformatics analysis of the proteomic analysis.

File Name: Supplementary Data 3
Description: Bioinformatics analysis on label-free quantitative mass spectrometry.

File Name: Supplementary Data 4
Description: Bioinformatics of small RNA sequencing analysis from Ago2 or Sfpq RNA-IP in RAW 264.7 cells.

File Name: Supplementary Data 5
Description: Bioinformatics assessment of the dismissal Ago2 binding activity upon Sfpq knockdown. Small RNA sequencing analysis of Ago2 HITS-CLIP experiments from P19 cells transfected with let-7a and siCtr, siSfpq and mimic Ctr, let-7a and siSfpq, or siCtr and mimic Ctr. Sfpq HITS-CLIP experiments were from P19 cells transfected with siSfpq or siCtr.

File Name: Supplementary Data 6
Description: Bioinformatics analysis of gene expression microarray analysis from P19 cells transfected with let-7a, siSfpq, let-7a+siSfpq, or control.

File Name: Supplementary Data 7
Description: Bioinformatics assessment of the recruitment Ago2 binding activity upon Sfpq knockdown. Bioinformatics of small RNA sequencing analysis from Ago2 experiments in P19 cells transfected with let-7a and siCtr, siSfpq and mimic Ctr, let-7a and siSfpq, or siCtr and mimic Ctr.

File Name: Supplementary Data 8
Description: Bioinformatics assessment of differential splicing events by SpliceTrap program from RNA-sequencing analysis of siSfpq-transfected P19 cells compared to siCtr-transfected ones.
